# Minimal important change and responsiveness of the Migraine Disability Assessment Score (MIDAS) questionnaire

**DOI:** 10.1186/s10194-021-01339-y

**Published:** 2021-10-21

**Authors:** Gabriela F. Carvalho, Kerstin Luedtke, Tobias Braun

**Affiliations:** 1 Institute of Health Sciences, Department of Physiotherapy, Pain and Exercise Research Luebeck (P.E.R.L), University of Luebeck, Ratzeburger Allee 160, 23562 Luebeck, Germany; 2 Laboratory of Pain Research, Institute of Physiotherapy and Health Sciences, The Jerzy Kukuczka Academy of Physical Education, Katowice, Poland; 3 IB University of Health and Social Sciences, Study Center Cologne, Cologne, Germany; 4 Department of Applied Health Sciences, Division of Physiotherapy, Hochschule für Gesundheit Bochum (University of Applied Sciences), Bochum, Germany

**Keywords:** Migraine, MIDAS, Disability, Measurement proprieties

## Abstract

**Background:**

The MIDAS is the most used questionnaire to evaluate migraine-related disability, but its utility to assess treatment response remains unclear. Our aim was to estimate the MIDAS’ minimal important change (MIC) value and its responsiveness.

**Methods:**

A total of 103 patients were enrolled in a non-pharmacological, preference-based clinical trial. MIDAS and global rating of self-perceived change (GRoC) scores were collected at baseline, after 5 weeks of treatment, 4-weeks and 3-months follow-up after treatment. Anchor-based approaches were used to establish MIC values and responsiveness.

**Findings:**

In all 3 timepoint comparisons, MIDAS presented a MIC of 4.5 points. A moderate positive correlation was identified between the MIDAS change and GRoC scores. The area under the curve ranged from 0.63 to 0.68.

**Conclusions:**

This study showed that MIDAS has a limited responsiveness to change. A change of 4.5 points or more represents a clinically important change for patients with high frequent migraine and chronic migraine receiving non-pharmacological treatment.

## Background

The Migraine Disability Assessment scale (MIDAS) [1] is the most frequently used questionnaire to assess migraine-related disability among patients with migraine, being used in at least 86 peer-reviewed publications in the last 5 years, including clinical trials [2]. It is recommended as an outcome measure of randomized clinical trials (RCTs) [3–5], since its items cover several relevant aspects suggested by the International Classification of Functioning, Disability, and Health (ICF) [2].

Although some measurement proprieties of the MIDAS, including validity and reliability, have been adequately evaluated [6], its utility to assess response to treatment remains unclear. According to the COSMIN [7] guidelines, estimation of an instrument’s responsiveness and other measurement proprieties such as minimal important change (MIC), are essential to assess a questionnaire’s ability to measure change over time (e.g., after a treatment). The minimal important change (MIC) is “the smallest change in a treatment outcome that an individual patient would identify as important and which would indicate a change in the patient’s management” [8]. MIC values can facilitate judgments of the magnitude of effect on patient reported outcomes. Therefore, the aim of this study is to estimate the MIC and responsiveness of the MIDAS questionnaire, along with its standard error of measurement (SEM) and smallest detectable change (SDC).

## Methods

This study is a secondary analysis of a published preference-based clinical trial, which aimed to assess the effectiveness of non-pharmacological interventions based on physiotherapy and aerobic exercise in patients with migraine [9]. A total of 103 patients with chronic migraine or frequent episodic migraine received physiotherapy (*n* = 79) or supervised aerobic exercise (*n* = 24) according to their preference as an add-on to the pharmacological treatment. As evidenced in the Table 1, the average age of the sample was 39.9 years (SD: 13.5), with an average headache frequency of 12.6 days per month (SD: 7.6) and headache were diagnosed on average 21 years ago (SD: 7.7). At baseline, patients had an average MIDAS scores of 20.2 (SD: 9.4) and for all data points ceiling and floor scores were less than 5% (Table 1).

**Table 1:** Sample characteristics exhibited as percentage (%), mean and standard deviation (SD)

	Baseline	Post-treatment	4-weeks follow-up	1-month follow-up
Age (years)	39.9 (13.5)			
Gender (%, male)	4%, 3			
Headache onset (years)	21.0 (7.7)			
Episodic migraine diagnosis (%, n) (days/month)	39.1%, 34 8.03 (3.25)			
Chronic migraine diagnosis (%) (days/month)	60.9%, 53 21.26 (5.15)			
Headache frequency (days/month)	12.6 (7.6)	10.5 (7.4)	10.2 (7.7)	9.2 (7.5)
MIDAS scores	20.2 (9.4)	12.8 (11.9)	13.6 (12.4)	13.3 (13.4)
MIDAS ceiling effect (%, n)	1%, 1	0%, 0	0%, 0	0%, 0
MIDAS floor effect (%, n)	3%, 3	2%, 2	5%, 4	3%, 2

The treatment duration was 5 weeks with two sessions of physiotherapy or aerobic exercise per week. Study outcomes were assessed at baseline, post-treatment, with follow-ups after 1 and 3 months after the last day of the intervention. Among selected primary and secondary outcomes, the MIDAS and the global rating of self-perceived change (GRoC) were included. Further details on study treatment and outcomes can be found in the published report [9].

The MIDAS questionnaire was developed to assess migraine-related disability over a 3-month recall period. It contains five questions regarding the number of days of missed work/school, reduced productivity at work/school, missed household work, reduced productivity in household work, and missed family and/or social activities. The total score is composed by the sum of the five items [1]. In the clinical trial, patients completed the questionnaire at all four timepoints using a one-month recall period for the MIDAS [9], as also suggested and employed in previous research trials [4, 5].

The GRoC scale was administered at the three timepoints following the treatment to assess the patients’ perception of their symptom change over time (post-treatment, follow-up at 1 and 3 months after the intervention). The score ranged from − 7 to + 7, with positive scores reflecting migraine improvement and negative scores reflecting worsening of the symptoms; a score of 0 indicated no change [10]. Patients who marked + 3 (somewhat better) or more were considered as having a significant improvement, while the ones who answered − 2 to + 2 or less were classified as unchanged [10]. Participants who scored − 3 or less were considered deteriorated and were excluded from the analysis due to the small number.

### Statistical analysis

According to the COSMIN study design checklist, a sample size of 50–99 subjects are adequate to assess the standard error of measurement (SEM), and greater than 50 subjects are very good to evaluate responsiveness [11]. The SEM was calculated based on the following formula: SEM = SD of MIDAS baseline x (SQR (1-ICC)). An intraclass correlation coefficient (ICC) of 0.99 was considered in the SEM calculation, based on a previously published reliability study [6]. The minimum detectable change (MDC) with 95% confidence was calculated based on the following formula: MDC_95_ = 1.96 × √2 × SEM.

The MIDAS change after treatment was calculated by subtracting the baseline scores from each of the other datapoints scores (post-treatment, 1 month follow-up and 3 months follow-up). Participants with missing MIDAS or GRoC data in one of the timepoints were excluded from the respective analyses. The GRoC scores were classified as “improved” (+ 3 to + 7) or “unchanged” (− 2 to + 2).

The anchor-based approach was used to assess the MIDAS’ responsiveness, as recommended by the COSMIN study design checklist [11]. The area under the receiver operating characteristic curve (AUC) was used to determine the probability to identify subjects who improved considering the complete dataset. An AUC ≥ 0.7 was considered satisfactory responsiveness [7]. Furthermore, Pearson’s correlation test was used to assess the correlation between absolute MIDAS score changes, migraine frequency change and GRoC scores for each of the timepoints [10].

An anchor-based approach (ROC analysis) was used to determine MIC values. As suggested by the COSMIN guidelines [7], the MIC cut-off point was chosen by selecting the point closest to the top left corner of the ROC curve, which represents the lowest overall misclassification between the “improved” and unchanged” participants. SPSS version 26.0 (IBM Corp.; Armonk, New York, USA) and Microsoft Excel 2019 (Microsoft Office; Redmond, Washington, USA) were used for data analysis and the alpha level was set at 5%.

## Results

**Fig. 1:**
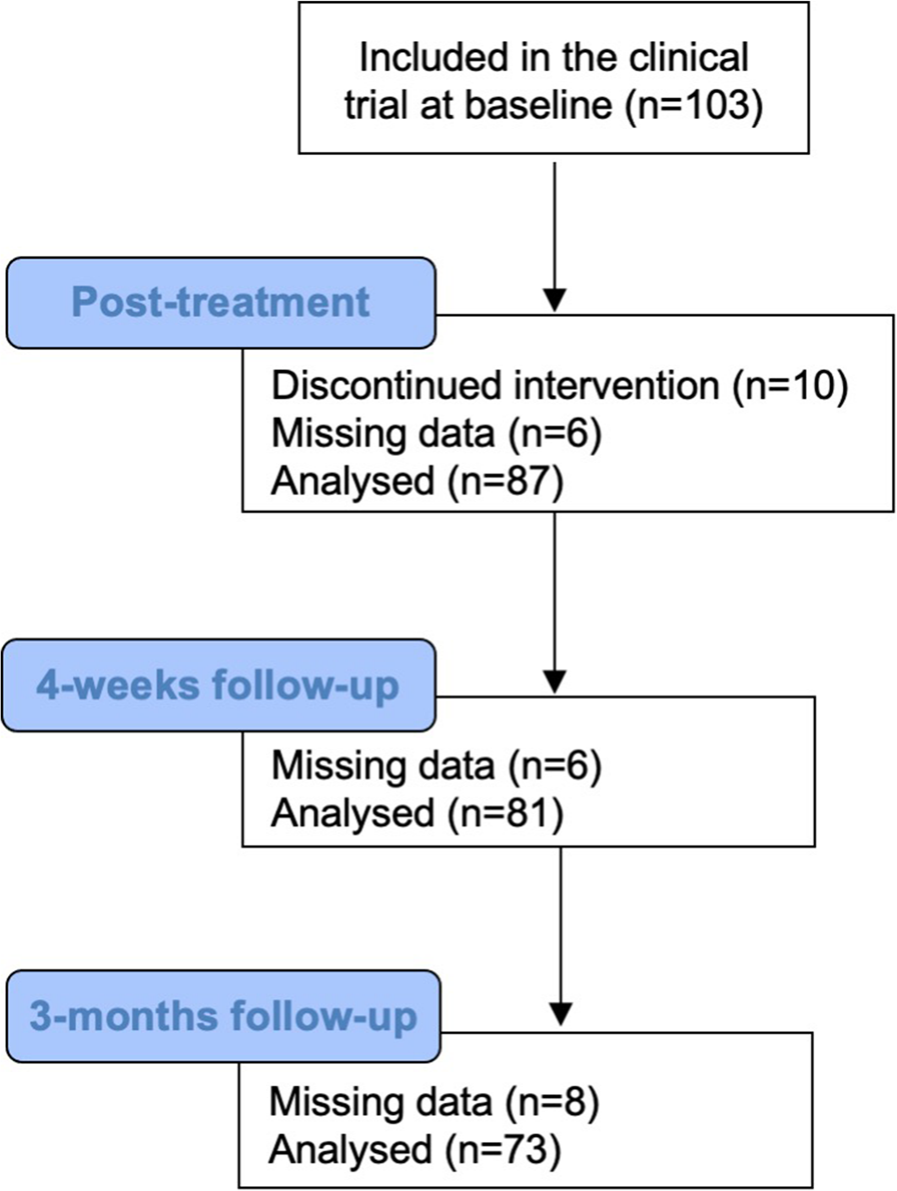
Study Flow Diagram

Considering the MIDAS scores at baseline, the questionnaire presented a SEM of 1.5 points and a MDC_95_ of 4.3 points. After excluding missing data, 87 patients were analyzed in the “pre *vs* post treatment”, 81 at “pre *vs* 4 weeks follow-up”, and 73 at “pre *vs* 3 months follow-up”. Missing data due to discontinuation of the trial are indicated in Fig. 1. In all three timepoint comparisons, the MIC of MIDAS was 4.5 points, with sensitivity values ranging from 0.58 to 0.67, and specificity values ranging from 0.61 to 0.62. A moderate positive and significant correlation was verified for MIDAS change and GRoC scores for all timepoints (*r* from 0.29 to 0.33). A strong positive and significant correlation was also found for absolute change of MIDAS scores and migraine frequency change for all timepoints (*r* = 0.90). In the timepoints “pre vs post treatment”, “pre vs 4 weeks follow-up”, “pre vs 3 months follow-up”, the AUCs were 0.68 (95% CI: 0.56 to 0.79), 0.65 (95% CI: 0.56 to 0.79), and 0.63 (95% CI: 0.49 to 0.76), respectively. All results can be found in Table 2.

**Table 2:** Minimal important change (MIC), sensitivity & specificity, responsiveness and Pearson’s correlation between migraine disability assessment scale **(**MIDAS) change scores, migraine frequency change and global rating scale of change (GRoC) after non-pharmacological treatment

	MIC (points)	Sensitivity/Specificity	Responsiveness (AUC^a^ and 95% CI)	Correlation MIDAS and GRoC	Correlation MIDAS and migraine frequency
Pre vs post treatment (*n* = 87)	4.5	0.67/0.62	0.68 (0.56 to 0.79)	0.32, *p* = 0.01	0.90, *p* < 0.0001
Pre vs 4-weeks follow-up (*n* = 81)	4.5	0.58/0.61	0.65 (0.56 to 0.79)	0.29, *p* = 0.01	0.90, *p* < 0.0001
Pre vs 3 months follow-up (*n* = 73)	4.5	0.64/0.62	0.63 (0.49 to 0.76)	0.33, *p* = 0.02	0.90, *p* < 0.0001

^a^Area under the curve

## Discussion

This study assessed, for the first time, the MIDAS’ responsiveness and MIC values, which are fundamental measurement proprieties for an adequate use of MIDAS in clinical research. Our results showed that MIDAS presents limited responsiveness to change, with a moderate correlation with GRoC scores. The minimal change considered important by patients is equal or more than 4.5 points, which was consistent for all 3 timepoint comparisons, and this value exceeds the MDC_95_ and SEM of the MIDAS.

Based on these results, sample size calculations based on MIC values can be performed using the MIDAS questionnaire, considering a 1 month recall period. However, it is important to highlight that although this form of administration is considered useful for clinical trials [3–5], the original recall period of the MIDAS is 3 months [1]. The measurement properties of the modified MIDAS, using a 1 month recall period, are unknown and need to be assessed. Using MIDAS based on 1 month recall could help to overcome one of the most important limitations of the questionnaire, the recall bias [3, 12]. In fact, patients with chronic migraine and medication overuse headache tend to report scores that are multiplied by 5 or 10 more frequently, in comparison with patients with episodic migraine [12]. Another reported limitation is that the 3-month recall MIDAS is often not correctly answered and should be simplified [3].

Despite the MIDAS limitations, it is considered a more valuable outcome assessment in comparison with headache frequency, since it reflects the disability and impact of the disease [3]. Furthermore, greater severity scores measured with MIDAS are related to greater eligibility to preventive migraine treatment [13], and greater sensory hypersensitivity, including presence of allodynia [14].

Regarding responsiveness, our results were slightly below to the 0.70-AUC cut-off point recommended by COSMIN, reflecting a limited questionnaire responsiveness [7]. However, the 95% confidence intervals of the three datapoint comparisons include the 0.70 cut-off value, and moderate correlations were observed between MIDAS change and patients’ perception of change over time.

Our results need to be considered in relation to the sample characteristics [9]. Since included patients presented chronic or high frequent episodic migraine, the MIC values are likely to be smaller than for patients with lower migraine frequency. Future studies are recommended to assess differences in MIDAS responsiveness among episodic and chronic patients. Furthermore, responsiveness is linked to a non-pharmacological treatment, which may be different for randomized clinical trials based on pharmacological interventions. In our sample, two different non-pharmacological modalities were adopted, and therefore it is possible that MIDAS responsiveness would differ among them. Despite these generalizability limitations, this study results are considered useful and relevant for both research and clinical settings when assessing disability in the migraine population.

## Key findings

- The minimal important change (MIC) of the MIDAS after non-pharmacological treatment of migraine is 4.5 points.
- The MIC of the MIDAS is larger than the measurement error, indicating clinical utility for the assessment of change in migraine associated disability.
- Limited responsiveness was verified for the MIDAS questionnaire with one-month recall period and needs to be assessed further.

## Data Availability

The datasets used and/or analyzed during the current study are available from the corresponding author on reasonable request.

## Abbreviations

COSMIN: Consensus-based standards for the selection of health measurement instruments
GroC: Global rating of self-perceived change
ICC: Intraclass correlation coefficient
ICF: International Classification of Functioning, Disability, and Health
MDC: Minimum detectable change
MIC: Minimal important change
MIDAS: Migraine disability assessment score
RCT: Randomized clinical trials
SDC: Smallest detectable change
SEM: Standard error of measurement
